# Induction of cancer-specific cytotoxicity towards human prostate and skin cells using quercetin and ultrasound

**DOI:** 10.1038/sj.bjc.6602364

**Published:** 2005-02-01

**Authors:** S Paliwal, J Sundaram, S Mitragotri

**Affiliations:** 1 Department of Chemical Engineering, University of California, Santa Barbara, CA 93106-5080, USA

**Keywords:** ultrasound, quercetin, heat shock protein, prostrate cancer, skin cancer, selective chemotherapy

## Abstract

Bioflavonoids, such as quercetin, have recently emerged as a new class of chemotherapeutic drugs for the treatment of various cancer types, but are marred by their low potency and poor selectivity. We report that a short application of low-frequency ultrasound selectively sensitises prostate and skin cancer cells against quercetin. Pretreatment of cells with ultrasound (20 kHz, 2 W cm^−2^, 60 s) selectively induced cytotoxicity in skin and prostate cancer cells, while having minimal effect on corresponding normal cell lines. About 90% of the viable skin cancer cell population was lost within 48 h after ultrasound-quercetin (50 *μ*M) treatment. Ultrasound reduced the LC_50_ of quercetin for skin cancer cells by almost 80-fold, while showing no effect on LC_50_ for nonmalignant skin cells.

Therapeutic selectivity plays a crucial role in determining the success of chemotherapy. Some of the current targeted therapies attempt to localise drugs to cancer cells based on overexpression of epidermal growth factor receptors (EGFR) (Mendelsohn and Baselga, 2000) or angiogenesis (Carter, 2001). Antibodies, inhibitors, antisense therapy and gene therapy are also among a few strategies that have gained momentum (Guillemard and Saragovi, 2004). Many of these strategies have now reached clinical trials; however, these methods are still limited by issues including low potency, delivery complications, multi-drug resistance, side effects, collateral damage (Tattersall and Clarke, 2003) or incomplete success (Lynch *et al*, 2004).

In an attempt to develop a targeted chemotherapeutic strategy, we propose the use of bioflavonoids, which are common dietary supplements, in conjunction with low-frequency ultrasound. Quercetin, a major bioflavonoid in human diet, has been identified as a chemotherapeutic agent for the treatment of breast cancer (Singhal *et al*, 1995; Choi *et al*, 2001), colon cancer (Salucci *et al*, 2002), ovarian cancer (Chan *et al*, 2003) and prostate cancer (Knowles *et al*, 2000; Nakanoma *et al*, 2001; Kobayashi *et al*, 2002). Antiproliferative action of quercetin is hypothesised to be mediated by attenuating phosphorylation of activated *hsp* transcription factor (*hsf*), shortly after its trimerisation (Nagai *et al*, 1995; Lee *et al*, 1998), thereby resulting in increased susceptibility of *hsf* to proteolytic degradation and as a consequence inhibiting all downstream events, including *hsp* expression (Li *et al*, 1999). Since *hsp*s are constitutively overexpressed in many tumours (Jaattela, 1999), inhibition of *hsp*s is an attractive chemotherapeutic strategy. *hsp*s form a complex with mutant p53 protein (mp53), thereby prolonging the half-life of malignant mp53 and allowing tumour cells to bypass the normal mechanism of cell cycle arrest (Selkirk *et al*, 1996).

In spite of its therapeutic benefits, utilisation of quercetin in clinical applications has been limited by low potency and poor specificity. Additionally, it is difficult to sustain therapeutic quercetin concentrations in blood by oral ingestion (Lamson and Bringall, 2000).

Here, through *in vitro* studies, we demonstrate for the first time, using two pairs of normal and cancer cells (human skin fibroblast and human prostate epithelial cells), that ultrasound selectively sensitises cancer cells against quercetin. LC_50_ of quercetin for skin cancer cells is selectively decreased by almost 80-fold by a short pretreatment with ultrasound.

## MATERIALS AND METHODS

### Cell culture

Normal and cancer cells derived from prostrate and skin tissues were investigated in this study. DU145 prostate cancer cells were provided by Dr L Wilson at UC Santa Barbara, CA, USA. Nonmalignant prostrate normal cells (Catalog No. CRL-11609), nonmalignant skin cells (Catalog No. CRL-7761) and skin cancer cells (Catalog No. CRL-7762) were obtained from the American Type Culture Collection (ATCC, Rockville, MD, USA). All cells were grown as monolayers and were kept in a 5% CO_2_ environment at 37°C. Cell cultures were maintained in Dulbecco's modified Eagle's medium (DMEM) with glucose (1 g l^−1^), NaHCO_3_ (3.7 g l^−1^), L-glutamine (2 mM), nonessential amino acids (0.0815 g l^−1^) and 10% FBS. Antibiotic–antimycotic cocktail (Catalog No. 15240-062, Gibco, Invitrogen Corporation, Carlsbad, CA, USA), at a final concentration of 100 U ml^−1^ of penicillin, 100 *μ*g ml^−1^ of streptomycin and 0.25 *μ*g ml^−1^ of amphotericin B, was added to all cultures. Cells were harvested at a concentration of about 3 × 10^5^ cells ml^−1^, by washing with versene (NaCl – 8 g l^−1^, KCl – 0.2 g l^−1^, NaH_2_PO_4_ – 1.15 g l^−1^, K_2_HPO_4_ – 0.2 g l^−1^, Na_2_-EDTA – 0.2 g l^−1^ in distilled water with pH adjusted to 7.2) followed by 2–3-min digestion with trypsin/EDTA (0.25%/0.02%).

### Ultrasound application and quercetin treatment

Aliquots of 2 ml cell suspension (3 × 10^5^ cells ml^−1^) were plated in 12-well plates. Ultrasound was applied to cells prior to quercetin exposure. Other sequences of ultrasound and quercetin application were not studied and may yield different results. Ultrasound was applied at a frequency of 20 kHz and an intensity of 2 W cm^−2^ (Sonics and Materials, Danbury, CT, model VCX 400). Ultrasound intensity was determined using a hydrophone (Tezel *et al*, 2002). Ultrasound was applied at room temperature for 60 s by directly immersing the transducer half-way down the meniscus in the well. The temperature of the cell suspension was recorded to ensure that no significant elevation of temperature (<5°C) was observed. 20 *μ*l of quercetin solution prepared in dimethyl sulphoxide (DMSO) was immediately added after each sonication to the wells to achieve a final quercetin concentration of 0–50 *μ*M. Cells were analysed for viability at the end of 48 h. In experiments involving multiple exposures of ultrasound, the adhering monolayer of cells in the wells was washed with the procedure described above. The washed cell suspension from each well was made up to 2 ml by adding DMEM and subsequently sonicated by plating it in a new 12-well plate. Additional exposures were performed in some experiments at the end of 48 and 72 h and cell viability assessed at the end of 96 h. Cell viability prior and during experimentation was determined using Trypan blue exclusion under a light microscope.

### Gel electrophoresis and Western blots

Malignant and nonmalignant skin cells were treated with ultrasound and quercetin (50 *μ*M) and their *hsp* content was assessed using Western blots after 48 h. Specifically, the culture medium was removed after the treatment and the wells were washed three times with PBS to remove the serum and dead cells. The removed culture medium was mixed with PBS and centrifuged for 10 min to recover the dead cell pellet. 200 *μ*l of lysis buffer containing 20 mM Tris (pH=7.4, Sigma), 150 mM NaCl (Sigma, St Louis, MO, USA), 1% Triton X-100 nonionic detergent buffer (ICN Biomedicals, Aurora, OH, USA) with 1 mM pepstatin, leupeptin and PMSF (Sigma Chemicals, St Louis, MO, USA) was added to the well and the dead cell pellet separately to obtain respective cell lysates. The cell lysates were then centrifuged for 10 min and the supernatant protein extracts were used for electrophoresis measurements. Electrophoresis samples were prepared on a cell number basis by mixing the two protein extracts and using the previously obtained cell density data, such that all the samples contained proteins from an equal number of starting cell population (roughly 6 × 10^5^ cells per well). Heat shock protein 72 (*hsp*72) mouse monoclonal IgG antibody (Catalog No: SPA-810, Stressgen, Victoria, BC, Canada) was used to measure the induction of inducible form of heat shock protein 70 family (*hsp*70), viz., *hsp*72. Anti-mouse IgG horseradish peroxidase-conjugated antibody (Amersham Pharmacia, UK) was used as the secondary antibody. Dilutions of 1 : 1000 for the primary antibody and 1 : 5000 for the secondary antibody were typically used. Images were captured using X-ray films by the ECL Western blotting detection kit (Amersham Pharmacia, UK) and quantified by densitometry by using the software ImageQuant™ TL (Amersham Biosciences, UK).

## RESULTS

**Figure 1:**
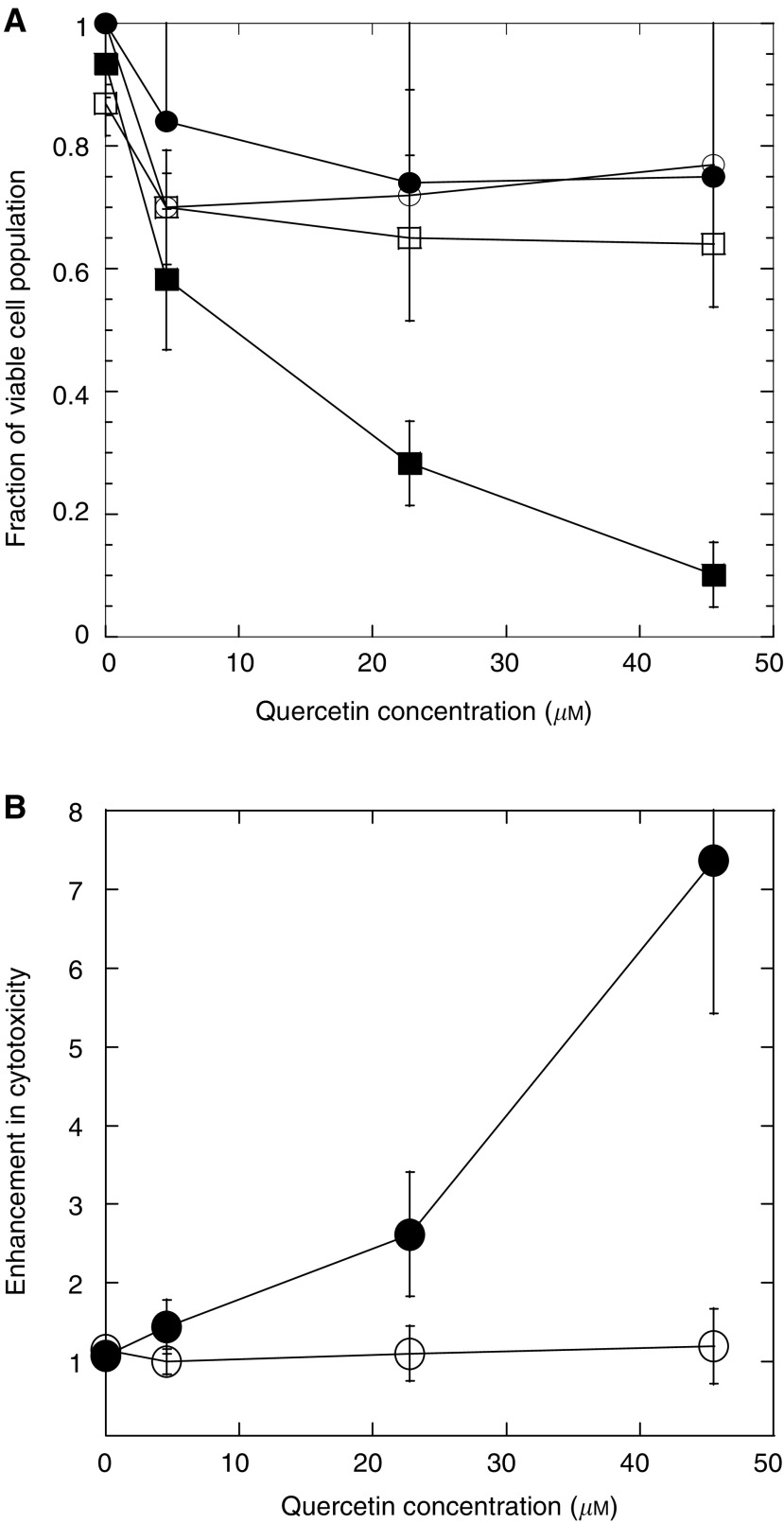
(**A**) Fractional loss of viable skin cancer cells (closed squares) and skin normal cells (open squares) when exposed to various concentrations of quercetin after a short exposure to ultrasound (20 kHz, 2 W cm^−2^, 60 s). *P*<0.25 for 5 *μ*M quercetin concentration; and *P*<0.001 for 25 and 50 *μ*M quercetin concentration. The figure also shows fractional loss of viable skin cancer cells (closed circles) and skin normal cells (open circles) when exposed to various concentrations of quercetin without ultrasound exposure. *P*>0.35 for 5 *μ*M quercetin concentration, and *P*>0.90 for 25 and 50 *μ*M quercetin concentration. Error bars indicate standard deviation. For skin cancer cells exposed to quercetin alone, error bars are shown only on one side for visual clarity. (**B**) Enhancement of cytotoxicity due to ultrasound application in skin cancer (closed circles) and skin normal cells (open circles) after incubation with quercetin at various concentrations. *P*<0.30 for 5 and 25 *μ*M quercetin concentrations; *P*<0.02 for 50 *μ*M quercetin concentration. Each point represents the average of three to five points. Error bars indicate standard deviation. Unpaired *t*-test for unequal variance was used to calculate probability values.

Cytotoxic effects of quercetin and ultrasound were assessed using two pairs of normal and cancer cell lines (human skin fibroblast and human prostate epithelial cells). The pair of skin cells was obtained from the same donor and differed from each other only in terms of malignancy. Cells were incubated with quercetin (0–50 *μ*M) with or without prior exposure to ultrasound (20 kHz, 2 W cm^−2^, 60 s). A strong concentration-dependent cytoxicity was observed in skin cancer cells for the combined ultrasound and quercetin treatment (Figure 1A, closed squares), but not in nonmalignant skin cells (Figure 1A, open squares, *P*<0.001 for quercetin=50 *μ*M). About 90% of viable population of skin cancer cells was lost in 48 h after ultrasound and quercetin (50 *μ*M) treatment (Figure 1A, closed squares). In the absence of ultrasound, quercetin showed no significant effect on either malignant or nonmalignant skin cells after 48 h incubation (Figure 1A, closed circles and open circles, respectively; *P*>0.90 for 50 *μ*M quercetin concentration). Similar results were obtained for prostate cancer and normal cells (data not shown, *P*<0.05 for ultrasound, followed by quercetin (50 *μ*M) treatment).

Enhancement in quercetin cytotoxicity towards skin cancer cells due to ultrasound exposure (defined as the fraction of cells killed with ultrasound exposure divided by the fraction of cells killed without the use of ultrasound at the same quercetin concentration) increased with increasing quercetin concentrations (Figure 1B, closed circles; *P*<0.02 for 50 *μ*M quercetin concentration). Ultrasound had no effect on quercetin toxicity towards nonmalignant skin cells (Figure 1B, open circles). Tumour selectivity (defined as the number of dead cancer cells divided by number of total dead cells; for equal number of normal and cancer cells treated) as high as 82% was observed. Ultrasound alone had no effect on cell viability of either type of skin cells (viability of 96±5% for both types of skin cells). The effect of ultrasound on quercetin-induced cytotoxicity is clearly due to the synergistic activity between the two and not due to the direct effect of ultrasound on cell viability.

**Figure 2:**
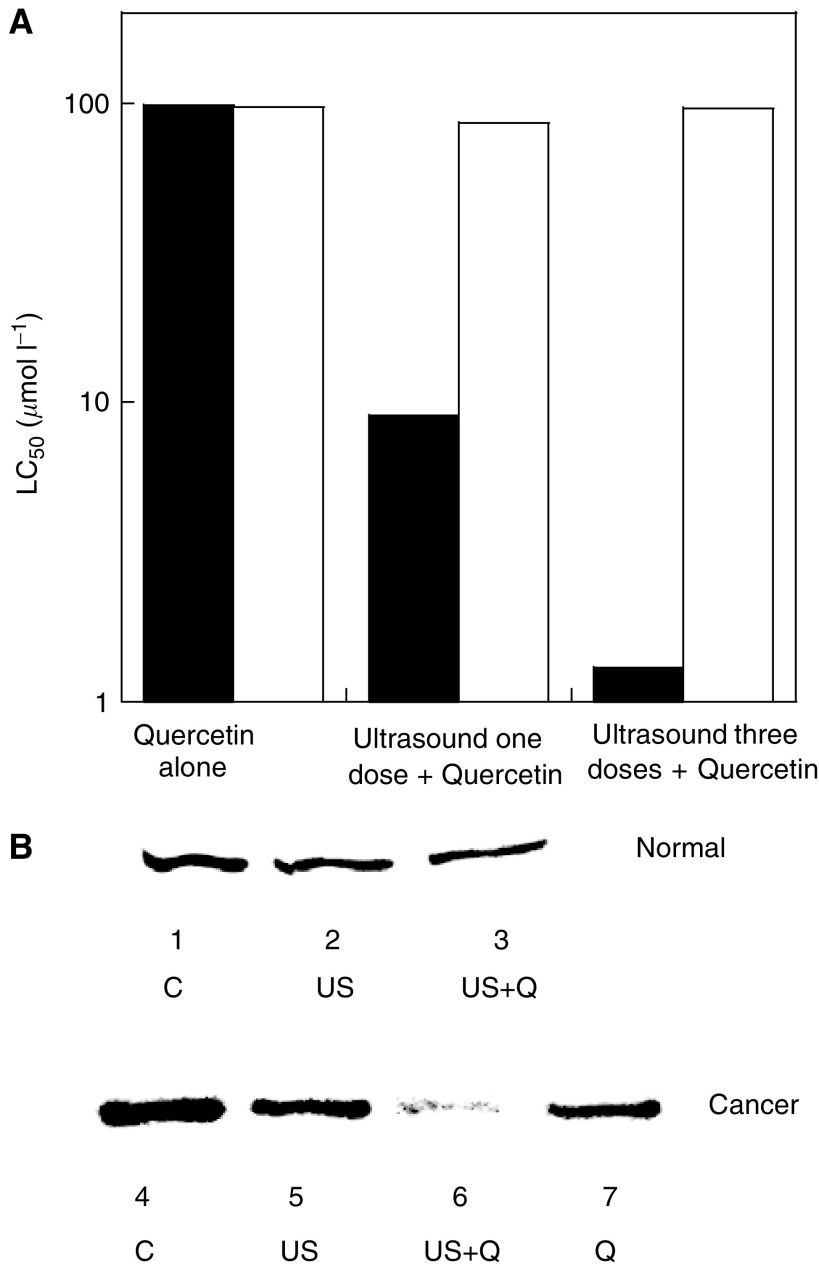
(**A**) Reduction of LC_50_ for skin cancer cells (filled bar) and skin normal cells (open bars) due to application of ultrasound and quercetin. Quercetin alone has an LC_50_ of about 98 *μ*M for skin cancer as well as skin normal cells. A single application of ultrasound (20 kHz, 2 W cm^−2^, 60 s) prior to incubation with quercetin substantially decreased LC_50_ for skin cancer cells to 9 *μ*M, but only moderately affected LC_50_ for skin normal cells (86 *μ*M). Application of three ultrasound pulses (prior to quercetin application, at 48 h and 72 h after the first application) further reduced LC_50_ for skin cancer cells to 1.2 *μ*M. Application of two pulses had no significant effect on LC_50_ for skin normal cells. (**B**) Cellular concentrations of *hsp*72 in nonmalignant skin cells (first three lanes) and skin cancer cells (last four lanes). The first lane shows *hsp*72 concentration in nonmalignant skin cells prior to exposure to ultrasound or quercetin (control). The second lane shows *hsp*72 concentration in nonmalignant cells exposed to ultrasound alone. The third lane shows *hsp*72 concentration in nonmalignant skin cells exposed to ultrasound, followed by 50 *μ*M quercetin for 48 h. The fourth lane shows control samples for skin cancer cells. *hsp*72 concentration in skin cancer cells is higher than that in skin normal cells. The fifth lane represents skin cancer cells exposed to ultrasound alone (20 kHz, 2 W cm^−2^, 60 s). The sixth lane shows *hsp*72 concentration in skin cancer cells exposed to ultrasound and subsequently to 50 *μ*M quercetin for 48 h. The seventh lane represents cells exposed to 50 *μ*M quercetin alone for 48 h.

The LC_50_ (quercetin concentration necessary to reduce cell viability by 50%) for skin cancer cells was also significantly reduced by ultrasound pre-exposure (Figure 2A: filled bar – skin cancer cells, open bar – nonmalignant skin cells). In the absence of ultrasound, LC_50_ of skin cancer cells was 98 *μ*M. However, a single exposure to ultrasound for 60 s reduced LC_50_ to about 9 *μ*M and two further applications of ultrasound 24 h apart reduced LC_50_ by 80-fold to about 1.2 *μ*M. LC_50_ of nonmalignant skin cells was not significantly altered (>50 *μ*M in all cases). To assess the specificity of synergy between quercetin and ultrasound, similar experiments were performed using another drug geldanamycin (a drug known to interfere with hsp90 cycle) and ultrasound. Geldanamycin alone exhibited cytotoxicity consistent with prior reports (Gan *et al*, 1998); however, no synergistic effect with ultrasound was found.

Selective effect of quercetin and ultrasound on skin cancer cells was accompanied by an effect on the inducible form of *hsp*70 (*hsp*72), which has long been known to confer protection to cells under severe stress (Kiang and Tsokos, 1998) and has been identified as a target of quercetin (Hansen *et al*, 1997). Skin cancer cells exhibited higher concentrations of *hsp*72 (1.8-fold, *P*<0.05) compared to corresponding nonmalignant cells (Figure 2B, lane 4 *vs* lane 1). This observation is consistent with the generally accepted notion that cancer cells overexpress heat shock proteins (Jaattela, 1999; Jolly and Morimoto, 2000; Nylandsted *et al*, 2000). Ultrasound alone or ultrasound+quercetin had minimal effect on cellular *hsp*72 in nonmalignant skin fibroblasts (Figure 2B, 12% decrease for ultrasound alone, *P*>0.90, and 22% decrease for ultrasound+50 *μ*M quercetin, *P*>0.76). A combination of ultrasound and quercetin (50 *μ*M) induced a significant decrease in *hsp*72 concentration in skin cancer cells (72% decrease, *P*<0.01, Figure 2B). In the same cells, quercetin alone decreased *hsp*72 concentration by 31.4% (Figure 2B, lane 7) and ultrasound alone decreased *hsp*72 concentration by 31.7% (Figure 2B, lane 5).

## DISCUSSION

The effects reported in Figures 1 and 2 are unlikely to originate from enhanced transport of quercetin by ultrasound. Quercetin is a small and slightly lipophilic molecule (molecular weight=302 Da, octanol–water partition coefficient, *K*_o/w_∼1.2±0.13 (Brown *et al*, 1998)) and is expected to diffuse across cell membranes at a high rate. Intracellular quercetin concentrations are expected to be in equilibrium with extracellular concentration even without ultrasound. Moreover, under the conditions used for the experiments in this study, a moderate degree of cavitation was observed (data not shown) and was not strong enough to induce significant membrane permeabilisation (as judged by lack of intracellular uptake of calcein under identical conditions), and hence incapable of pushing quercetin into cells.

It is not clear at this stage as to how ultrasound selectively sensitises cancer cells against quercetin. It is possible that the selectivity originates from the effect of quercetin as well as ultrasound on stress response. Quercetin has been shown to interfere with the stress response and inhibit *hsp*72 both at protein and mRNA levels in certain cells (Hosokawa *et al*, 1990; Elia and Santoro, 1994; Jakubowicz-Gil *et al*, 2002). Ultrasound, being a mild stress, may also induce a stress response in mammalian cells. It is possible that the interplay between the effect of ultrasound and quercetin on *hsp* cycle leads to selective sensitisation of cancer cells against ultrasound. Whether or not other mild stresses, for example, hypoxia, yield similar results remains to be seen. Since elevated levels of *hsp*s are broadly associated with survival of cancer cells (Burdon, 1987; Lasunskaia *et al*, 1997), chemotherapeutic strategies that target *hsp*s are attractive. With further studies focused on *in vivo* testing and mechanistic understanding, this technique may provide a potential treatment for the treatment of cancer, especially skin cancer.
